# Cluster analysis of extracellular matrix biomarkers predicts the development of impaired systolic function within 1 year of acute myocardial infarction

**DOI:** 10.1007/s00380-022-02118-8

**Published:** 2022-07-27

**Authors:** Morgane M. Brunton-O’Sullivan, Ana S. Holley, Bijia Shi, Scott A. Harding, Peter D. Larsen

**Affiliations:** 1 Department of Surgery and Anaesthesia, The University of Otago, Wellington, New Zealand; 2 Wellington Cardiovascular Research Group, The University of Otago, Wellington, New Zealand; 3 School of Biological Sciences, Victoria University of Wellington, Wellington, New Zealand; 4 Department of Cardiology, Wellington Regional Hospital, Wellington, New Zealand

**Keywords:** Acute myocardial infarction, Extracellular matrix, Biomarkers, Combined biomarker analysis, Cluster analysis

## Abstract

The clinical utility of combining extracellular matrix (ECM) biomarkers to predict the development of impaired systolic function following acute myocardial infarction (AMI) remains largely undetermined. A combination of ELISA and multiplexing assays were performed to measure matrix metalloproteinase (MMP)-2, MMP-3, MMP-8, MMP-9, periostin, N-terminal type I procollagen (PINP) and tissue inhibitor of matrix metalloproteinase-1 (TIMP-1) in plasma samples from 120 AMI patients. All patients had an echocardiogram within 1 year of AMI, and were divided into impaired (*n* = 37, LVEF < 50%) and preserved (*n* = 83, LVEF ≥ 50%) systolic function groups. Exploratory factor analysis was performed on log-transformed biomarkers using principle axis analysis with Oblimin rotation. Cluster analysis was performed on log-transformed and normalised biomarkers using Ward’s method of minimum variance and the squared Euclidean distance metric. Upon univariate analysis, current smoking, prescription of ACE inhibitors at discharge, peak hsTnT > 610 ng/L (median), MMP-8 levels, Factor 1 scores and Cluster One assignment were predictive of impaired systolic function. Upon multivariate analysis, Cluster One assignment (odds ratio [95% CI], 2.74 [1.04–7.23], *p* = 0.04) remained an independent predictor of systolic dysfunction in combination with clinical variables. These observations support the usefulness of combining ECM biomarkers using cluster analysis for predicting the development of impaired systolic function in AMI patients.

**Supplementary Information:**

The online version contains supplementary material available at 10.1007/s00380-022-02118-8.

## Introduction

A major consequence of acute myocardial infarction (AMI) is the subsequent development of adverse left ventricular (LV) remodelling. Described by progressive changes to LV geometry and function, adverse remodelling is closely associated with increased risk of poor cardiovascular outcomes [1] and persists following AMI despite optimal medical therapy and clinical management [2, 3]. Systolic dysfunction is a clinical manifestation of adverse LV remodelling and is characterised by reduced left ventricular ejection fraction (LVEF) [4]. While commonly presenting as asymptomatic in patients, systolic dysfunction is a significant risk factor for the development of heart failure with reduced ejection fraction (HFrEF) [5].

The cardiac extracellular matrix (ECM) is comprised of multiple molecular factors that provide structural support to the myocardium and facilitate cardiac repair processes following ischaemic injury [6]. Collagen deposition is central for stable scar formation and is critical for the development of cardiac fibrosis [7]. While localised to the myocardium, by-products of collagen maturation can be measured as biomarkers of collagen synthesis, such as N-terminal type I procollagen (PINP). In addition, matrix metalloproteinases (MMPs) and their endogenous regulators, tissue inhibitors of matrix metalloproteinases (TIMPs), are important proteolytic enzymes involved in remodelling the architecture of the myocardium following injury [8]. Matricellular proteins, such as periostin, have a limited role in matrix architecture, and instead facilitate matrix remodelling processes by acting as an interface between the cellular and ECM network [9]. Indeed, a number of candidate ECM biomarkers have been implicated as prognostic tools for the development of systolic dysfunction [10–13] and heart failure [14, 15]. However, current studies are mostly limited to single biomarker analysis and inconsistencies of prognostic effectiveness exist within the literature. Potentially, investigating strategies to combine multiple biomarkers may more optimally capture cardiac remodelling, which is characterised as a complex pathophysiological process.

We have previously demonstrated that combining biomarkers using cluster analysis can separate AMI patients into groups with differential risk based on Global Registry of Acute Coronary Events (GRACE) scores and peak high-sensitivity Troponin T (hsTnT) levels [16]. Cluster analysis describes the partitioning of similar objects together and dissimilar objects apart using the expression of input variables, such as biomarker levels [17]. Subsequently, cluster analysis is a powerful tool to investigate patient risk based on the combined expression of multiple biomarkers. In our previous study, we also utilised exploratory factor analysis (EFA) to examine the underlying interrelationships between ECM biomarkers [16]. In addition to this purpose, factor scores can also be generated from EFA as a mechanism to investigate the relationship between combined variables and patient risk [18]. Thus, cluster analysis and EFA are two techniques that can be applied to a clinical population to investigate the utility of combining biomarkers.

The aim of this study was to assess if combining ECM biomarkers using EFA or cluster analysis could more accurately predict the development of impaired systolic function in AMI patients when compared to single biomarker analysis. Seven ECM biomarkers were examined in this study based on previously published findings that suggest MMP-2, MMP-3, MMP-8, MMP-9, periostin, PINP and TIMP-1 were an optimal biomarker panel to assess prognostic outcomes in AMI [19].

## Materials and methods

### Study population

Patients diagnosed with acute coronary syndromes (ACS) and undergoing coronary angiography with or without percutaneous coronary intervention at Wellington Regional Hospital between January 2012 and September 2018 were prospectively recruited into the Wellington ACS Registry. ACS was defined as having symptoms suggestive of myocardial ischaemia for greater than 10 min alongside either troponin elevation or ≥ 1 mm of new ST-segment deviation or T wave inversion as identified using an electrocardiogram in a minimum of two contiguous leads [20]. Patients were excluded from this registry if they had a platelet count less than 100 × 10^9^ /L, a known platelet function disorder or were administered a fibrinolytic agent within 24 h or a glycoprotein IIb/IIIa receptor agent within a week of enrolment. From this cohort, a subset of 120 AMI patients were selected who had no previous history of myocardial infarction or atrial fibrillation and who had echocardiogram measurements at a remote time point within 1 year from index admission. This study was approved by the Lower South Regional Ethics Committee (LRS/11/09/035) and the New Zealand Central Health and Disabilities Ethics Committee (16/CEN/68).

### Data collection and blood sampling

Clinical characteristics were prospectively collected upon review of medical records. Whole blood was collected into sodium citrate tubes (0.109 M, BD Vacutainer, New Jersey, USA) either from a peripheral vein using a 21-gauge needle before angiography or from the arterial sheath during cardiac catheterization prior to heparin administration. Citrated whole blood was separated into plasma by centrifugation at 1500 × g for 12 min, and aliquots were stored at  – 80 °C for subsequent analysis. Cardiac specific peak hsTnT measurements were recorded for all AMI patients as part of routine standard of care using the Elecsys Troponin T high-sensitive test (Roche Diagnostics, Basel, Switzerland).

### Echocardiogram assessment

A 2-dimensional transthoracic echocardiogram was performed on each patient by a trained sonographer during clinical follow-up within 1 year of AMI onset. A standard transthoracic echocardiographic imaging protocol was used [21], with apical four-chamber and two-chamber views; LV short axis were recorded from the left parasternal region at the following three levels: the mitral valve, the mid-papillary muscle, and the apex. All calculations and interpretations of echocardiogram reports were performed by cardiologists or cardiac sonographers, and missing values identified upon retrospective analysis were completed by an advanced cardiology trainee. Systolic function was categorised into preserved and impaired function based on the updated recommendations by the American Society of Echocardiography and the European Association of Cardiovascular Imaging (ASE/EACVI) [22]. Preserved function was defined as LVEF ≥ 50% and impaired function was defined as LVEF < 50%.

### Biomarker measurement

The biomarker panel selected for this study comprised the following: MMP-2, MMP-3, MMP-8, MMP-9, periostin, PINP and TIMP-1. In a previous study, we demonstrated that these ECM biomarkers were of interest to investigate in a prognostic setting following AMI [19]. Specifically, we showed that levels of these biomarkers were significantly altered in AMI patients when compared to healthy volunteers and that these biomarkers were stably expressed in the first 3 days following symptom onset, confirming opportunistic blood sampling during acute hospital admission. Biomarker levels were quantified in plasma samples of ACS patients blinded to systolic function categorisation. The levels of all MMPs and periostin were measured using two Magnetic Luminex panels according to manufacturer instructions. Panel One contained MMP-2, MMP-3 and MMP-9 (Lot L136374, 1:25 sample dilution) and Panel Two comprised MMP-8 and Periostin (Lot L125986, 1:2 sample dilution). The mean fluorescence intensity for each analyte was measured using dual-lasers on the Luminex 200 analyzer (Sigma-Aldrich, Massachusetts, USA). Experimental data were analysed by fitting a 5-PL curve to the standard analyte curves. Intra-assay coefficients of variation ranged between 4.4% and 7.2%, and inter-assay coefficients of variation were between 4.5% and 14.1%. Levels of PINP (Human PINP ELISA, MyBioSource, California, USA) and TIMP-1 (Human Duoset ELISA, R&D Systems, Minnesota, USA) were measured using ELISA according to manufacturer instructions. Optimal density was measured at 450 nm, with a wavelength correction set to 570 nm. 4-PL and 5-PL standard curves were generated to determine sample concentrations of TIMP-1 and PINP, respectively. Intra-assay coefficients of variation ranged between 3.4% and 10.0%, and inter-assay coefficients of variation were between 2.9% and 7.4%.

### Statistical analysis

Continuous variables were assessed for normality using the Shapiro–Wilk test. Parametric continuous variables were reported as mean ± standard deviation (SD) and non-parametric continuous variables were reported as median (interquartile range; IQR). Categorical variables were reported as frequencies (percentages). Mann–Whitney U or Kruskal–Wallis *H* Test was undertaken to compare continuous and categorical variables. Chi Square testing was utilised to compare categorical variables. To construct multivariate regression modelling, all demographics and clinical characteristics with a *p*-value < 0.05 on univariate analysis were included in binary logistic regression analysis. Statistical significance was determined in this study by *p* < 0.05. All basic statistical analysis were carried out in either GraphPad Prism Software v.7.04 (GraphPad Software Inc; California, USA) or SPSS v.24 (IBM; New York, USA).

EFA and cluster analysis were performed as previously described [16]. Briefly, EFA was performed on seven log-transformed ECM biomarkers using principle axis analysis with Oblimin rotation using SPSS v.24. Eigenvalues > 1 and parallel analysis were performed to confirm factor number. Parallel analysis was performed using an online engine [23]. All variables with factor loadings > 0.3 were presented in this study. Cluster analysis was performed exclusively on ECM biomarker data and did not include clinical characteristics or patient risk factors. Prior to cluster analysis, biomarker data were log-transformed to normalise distribution and each biomarker was standardised to the same scale (mean = 0, SD = 1) to account for large variance between biomarkers which could influence cluster assignment. Subjects were partitioned using agglomerative hierarchical clustering using Ward’s method of minimum variance and the squared Euclidean distance metric in R version 4.0.2 [24]. Identification of optimal cluster number was assessed using two clustering indices measured using the NbClust package [25] and visualised using the Factoextra package [26] in R version 4.0.2 [24]. A cluster size of two was determined as optimal for this study based on Average Silhouette Width (Supplementary Fig. 1) and the ‘Within Sum of Squares’ methodology (Supplementary Fig. 2).

## Results

### Clinical characteristics

A summary of the clinical characteristics and levels of ECM biomarkers for the patient population is shown in Table 1. There were 83 patients (69.2%) with preserved systolic function and 37 patients (30.8%) with impaired systolic function within 1-year of AMI onset. When these groups were compared, patients with impaired systolic function were less likely to be current smokers (5.4% versus 24.1%, *p* < 0.05), more commonly discharged on angiotensin-converting enzyme (ACE) inhibitor medication (83.8% versus 60.2%, *p* < 0.05) and had higher levels of peak hsTnT (median 2174.5 [IQR 561.3–4184.8] ng/L versus 499.0 [163.0–1580.0] ng/L). Time to echocardiogram was similar between systolic function groups, with a median time of 145 days (IQR 88–251) post-symptom onset. Of the seven ECM biomarkers, only MMP-8 levels were significantly different between patient groups and were elevated in patients with impaired systolic function compared to preserved systolic function (0.56 [0.49–0.71] ng/mL versus 0.51 [0.48–0.60] ng/mL, p < 0.05).

**Table 1:** Baseline demographics of patients with preserved and impaired systolic function

Baseline demographics	Total cohort (*n* = 120)	Preserved systolic function (*n* = 83)	Impaired systolic function (*n* = 37)	*P* value
Male	94 (78.3)	62 (74.7)	32 (86.5)	0.148
Age (years)	62 ± 11	62 ± 12	61 ± 10	0.517
BMI	29.2 ± 5.8	28.7 ± 5.6	30 ± 6.5	0.320
*Ethnicity*				
European	94 (78.3)	65 (78.3)	29 (78.4)	0.238
Māori	11 (9.2)	6 (7.2)	5 (13.5)	
Pacific Islander	4 (3.3)	2 (2.4)	2 (5.4)	
Asian, Indian & Latin	11 (9.2)	10 (12.0)	1 (2.7)	
*Cardiac risk factors*				
Hypertension	62 (51.7)	46 (55.4)	16 (43.2)	0.218
Dyslipidaemia	59 (49.2)	41 (49.4)	18 (48.6)	0.940
Diabetes	22 (18.3)	18 (21.7)	4 (11.1)	0.172
Current smoker	22 (18.3)	20 (24.1)	2 (5.4)	**0.015**
Renal dysfunction	2 (1.7)	1 (1.2)	1 (2.7)	0.554
*AMI Classification*				
NSTEMI	76 (63.3)	57 (68.7)	19 (51.4)	0.069
STEMI	44 (36.7)	26 (31.3)	18 (48.6)	
*Discharge medications*				
Aspirin	100	100	100	–
P2Y12 inhibitor	100	100	100	–
ACE Inhibitor	81 (67.5)	50 (60.2)	31 (83.8)	**0.011**
Beta blocker	96 (80.0)	66 (79.5)	30 (81.1)	0.843
CA Channel inhibitor	8 (6.7)	6 (7.2)	2 (5.4)	0.700
Statins	113 (94.2)	79 (95.2)	34 (91.9)	0.478
*Echocardiogram features*				
LVESV (mL)	53.2 (35.4–72.8)	40.6 (28.4–55.0)	79.0 (64.8–97.7)	** < 0.0001**
LVEDV (mL)	115.4 (93.6–139.5)	107.8 (81.1131.8)	139.2 (115.3–166.6)	** < 0.0001**
*Clinical parameters*				
Time to echocardiogram (days)	145 (89–252)	143 (80–245)	163 (91–296)	0.341
Peak hsTnT (ng/L)	610.0 (214.0–2442.0)	499.0 (163.0–1580.0)	2174.5 (561.3–4184.8)	** < 0.001**
*ECM biomarkers (ng/mL)*				
MMP-2	110.7 (98.2–122.3)	110.8 (98.6–123.1)	109.9 (94.1–120.5)	0.576
MMP-3	9.6 (7.1–14.2)	9.0 (7.0–13.8)	9.7 (7.3–16.5)	0.493
MMP-8	0.52 (0.49–0.63)	0.51 (0.48–0.60)	0.56 (0.49–0.71)	**0.047**
MMP-9	17.8 (12.8–29.8)	17.5 (12.2–26.2)	21.5 (14.0–38.9)	0.141
Periostin	64.3 (48.3–74.4)	64.7 (51.8–75.6)	61.4 (43.9–71.9)	0.205
PINP	42.2 (34.3–56.4)	42.2 (34.3–57.5)	43.7 (34.3–54.5)	0.748
TIMP-1	81.0 (72.3–95.7)	80.2 (70.6–93.2)	87.1 (75.8–116.0)	0.097

Continuous variables are expressed as median (IQR). Mann-Whitney U testing was used to compare continuous variables between clustered groups. Categorical variables are expressed as frequencies (percentages). Chi-Square testing was used to compare categorical variables between clustered groups

Abbreviations: *BMI* body mass index; *NSTEMI* non-ST elevation myocardial infarction; *STEMI* ST-elevation myocardial infarction; *LVESV* left ventricle end-systolic volume; *LVEDV* left ventricle end-diastolic volume; *hs* high-sensitivity; *ACE* angiotensin-converting enzyme; *CA* calcium; *MMP* matrix metalloproteinase; *PINP*, procollagen type I N-terminal propeptide, *TIMP*, tissue inhibitor of matrix metalloproteinase; *hsTnT* high-sensitivity Troponin T

Significant p-values are bolded (*p* < 0.05)

### EFA analysis of ECM biomarkers

EFA was performed on log-transformed biomarkers using principle axis analysis with Oblimin rotation. Model fit was assessed using Kaiser–Meyer–Olkin (KMO) and was above the required threshold of 0.5 with a value of 0.6. Collinearity of the dataset was assessed using Bartlett’s Test for Sphericity and was found to be significant (*p* < 0.0001). Factor loadings were suppressed below a 0.3 threshold to only include meaningful variables.

**Fig. 1:**
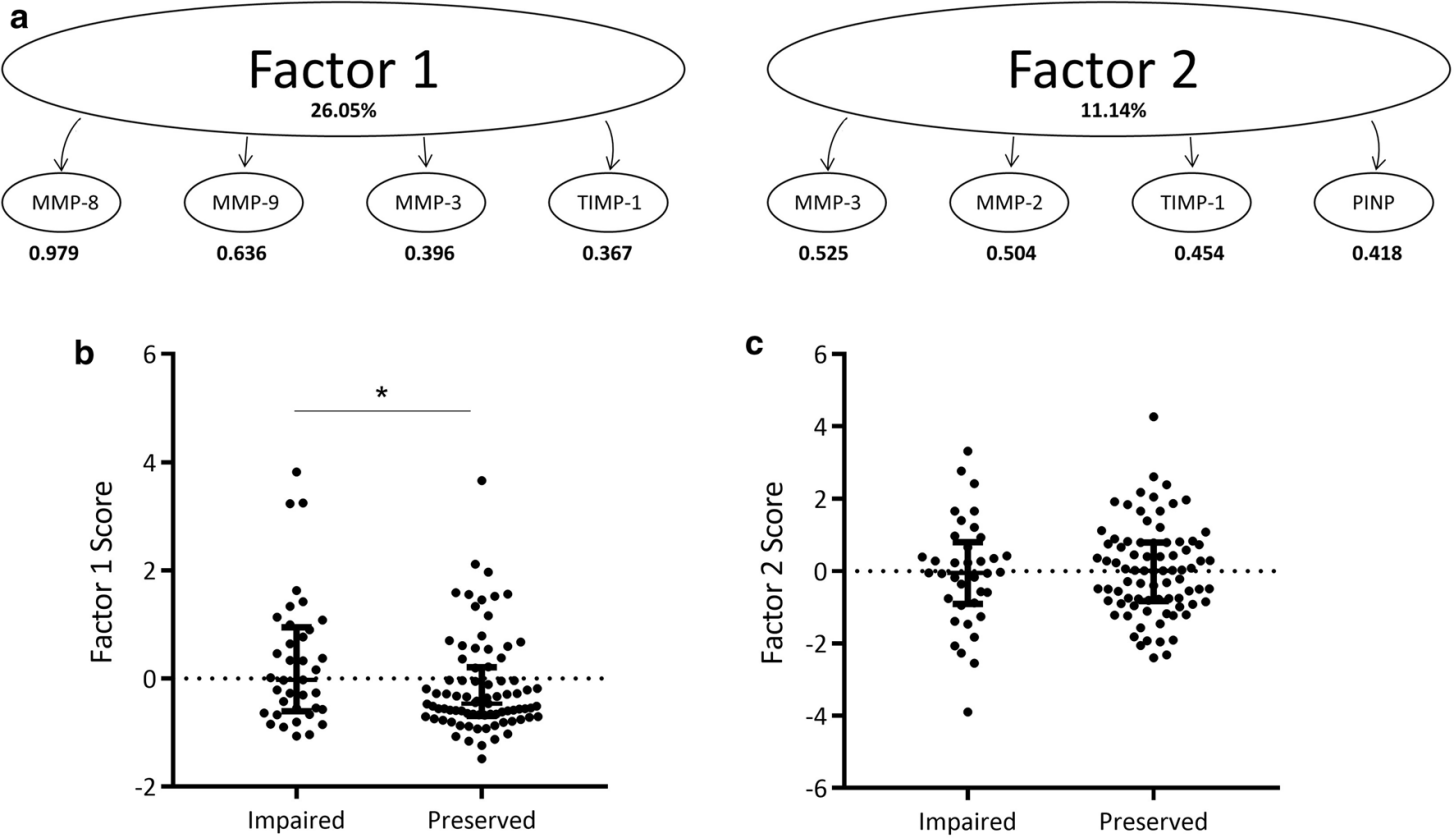
Exploratory factor analysis results in cohort of AMI patients** a** The rotated factor matrix of log-transformed ECM biomarkers in 120 AMI patients using EFA. The large ovals represent each latent factor with the percentage of variance for each factor described in bold. The small ovals represent the variables included within each latent factor, and the loading factors are displayed in bold below. **b** Mann–Whitney *U* testing demonstrated a significant increase in Factor 1 scores in patients with impaired systolic function compared to preserved systolic function. **c** No differences were observed in Factor 2 scores between systolic function groups. Median and interquartile range are plotted, and graphs were created using GraphPad Prism software, version 7.04 for Windows. Abbreviations: *MMP* matrix metalloproteinase, *TIMP* tissue inhibitor of matrix metalloproteinase, *PINP* N-terminal type I procollagen

EFA identified a two-factor solution, and the rotated factor matrix is shown in Fig. 1a. Factor 1 comprised of MMP-8 and MMP-9 with high loading values > 0.6, and MMP-3 and TIMP-1 with lower loading factors. Factor 2 comprised of MMP-3, MMP-2, TIMP-1 and PINP all with moderate loading factors between 0.4 and 0.5. MMP-3 and TIMP-1 were cross-correlated between factors. Both factors had higher loadings on Factor 2, suggesting they contributed to the composition of this factor more than Factor 1. Despite being included in the EFA analysis, periostin did not contribute to either factor. Factor scores were generated for each patient and these scores were compared between preserved and impaired systolic function groups. Factor 1 scores were significantly elevated in patients with impaired systolic function compared to patients with preserved function ( – 0.02 [ – 0.60 to 0.95] versus  – 0.46 [ – 0.69 to 0.21], *p* < 0.05; Fig. 1b). No differences in Factor 2 scores were observed between patient groups (Fig. 1c).

### Cluster analysis of ECM biomarkers

**Fig. 2:**
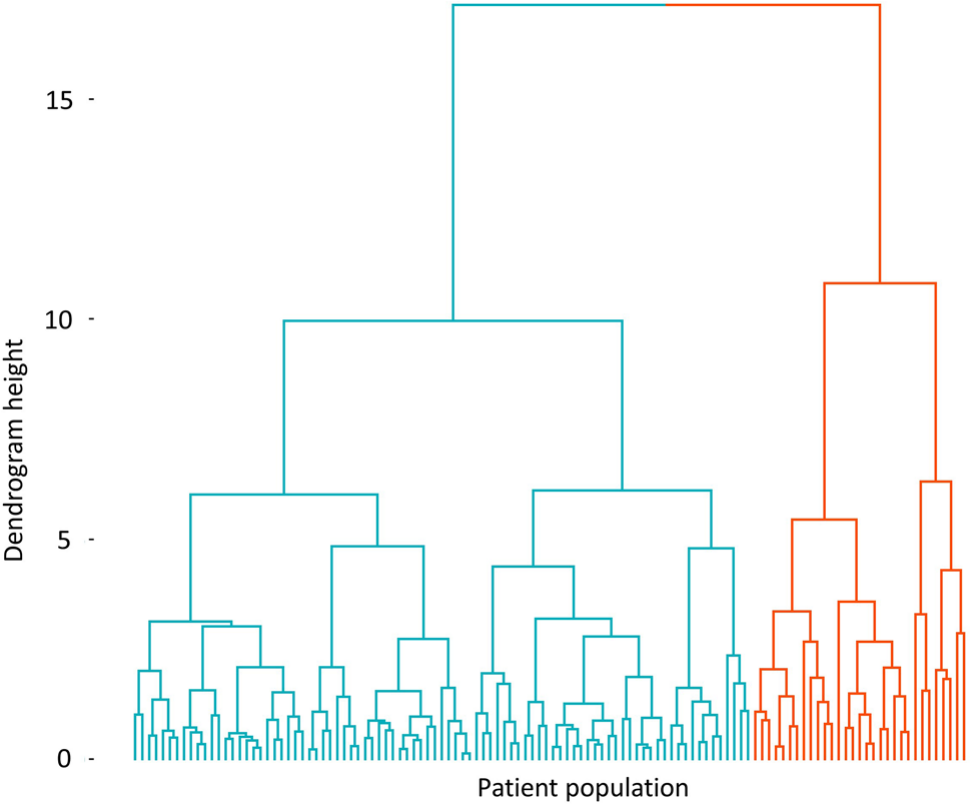
Dendrogram of cluster analysis performed on the AMI population. Hierarchical cluster analysis performed using MMP-3, MMP-8, MMP-9 and TIMP-1 biomarker levels separated patients into two distinct groups. Cluster One (*n* = 83) is shown in blue, and Cluster Two (*n* = 37) is shown in orange. This image was created using the factoextra package in R version 4.0.2, www.R-project.org

Factor 1 scores derived from EFA analysis were significantly elevated in patients with impaired systolic function compared to preserved function. Consequently, hierarchical cluster analysis was performed using the biomarkers that encompassed this factor which included the following: MMP-3, MMP-8 and MMP-9, TIMP-1. Cluster analysis identified two patient groups (Fig. 2). Differences in clinical characteristics and ECM biomarker levels between impaired and preserved systolic function patients are shown in Table 2.

**Table 2:** Baseline demographics of clustered groups

Baseline demographics	Cluster one (*n* = 31)	Cluster two (*n* = 89)	*P* value
Male	**29 (93.5)**	**65 (73.0)**	**0.017**
Age (years)	61 ± 11	62 ± 12	0.362
BMI	29.6 ± 6.4	29.0 ± 6.4	0.783
*Ethnicity*			
European	24 (77.4)	70 (78.7)	0.999
Māori	3 (9.6)	8 (9.0)	
Pacific Islander	1 (3.2)	3 (3.4)	
Asian, Indian, Latin	3 (9.6)	8 (9.0)	
*Cardiac risk factors*			
Hypertension	16 (51.6)	46 (51.7)	0.994
Dyslipidaemia	15 (48.4)	44 (49.4)	0.920
Diabetes	7 (22.6)	15 (16.9)	0.495
Current Smoker	5 (16.1)	17 (19.1)	0.713
Renal Dysfunction	0 (0)	2 (2.2)	0.400
*AMI Classification*			
NSTEMI	21 (67.7)	55 (61.8)	0.554
STEMI	10 (32.3)	34 (38.2)	
*Clinical parameters*			
Time to echocardiogram (days)	176.0 (96.0–328.0)	139.0 (84.0–244.5)	0.156
Impaired systolic function (n)	15 (48.4)	24 (24.7)	** < 0.05**
Peak hsTnT (ng/L)	2049.0 (490.0–3468.0)	539.5 (158.0–2118.3)	** < 0.001**
*ECM biomarkers (ng/mL)*			
MMP-2	111.8 (96.2–119.4)	109.9 (98.8–122.8)	0.706
MMP-3	10.9 (8.4–17.2)	9.0 (6.4–13.4)	**0.015**
MMP-8	0.78 (0.63–0.84)	0.50 (0.47–0.54)	** < 0.0001**
MMP-9	48.5 (33.1–64.4)	15.6 (11.8–19.7)	** < 0.0001**
Periostin	61.7 (42.6–67.7)	65.0 (52.5–76.1)	0.074
PINP	47.1 (35.1–56.5)	42.2 (34.3–57.6)	0.700
TIMP-1	87.9 (72.7–124.0)	80.0 (72.0–93.9)	0.164

Continuous variables are expressed as median (IQR). Mann-Whitney U testing was used to compare continuous variables between clustered groups. Categorical variables are expressed as frequencies (percentages). Chi-Square testing was used to compare categorical variables between clustered groups

Abbreviations: *BMI* body mass index; *NSTEMI* non-ST elevation myocardial infarction; *STEMI* ST-elevation myocardial infarction; *LVEF* left ventricular ejection fraction; *LVESV* left ventricle end-systolic volume; *LVEDV* left ventricle end-diastolic volume; *hsTnT* high-sensitivity Troponin T

Significant p-values are bolded (*p* < 0.05)

Patients in Cluster One (*n* = 31) had decreased LVEF measurements (*p* < 0.05) and elevated levels of MMP-3 (*p* < 0.05), MMP-8 (*p* < 0.0001) and MMP-9 (*p* < 0.0001) compared to Cluster Two (*n* = 89). Of the clinical characteristics assessed in this study, only gender was significantly different between clustered groups with more male patients in Cluster One compared to Cluster Two (*p* < 0.05).

### Multivariate analysis

Binary logistic regression analysis was undertaken to investigate the predictive potential of MMP-8 levels, Factor 1 scores and cluster assignment for identifying systolic dysfunction in AMI patients. The prescription of ACE inhibitors at discharge, current smoking status and peak hsTnT > median were also predictors of systolic function and were, therefore, included in the multivariate model. As Factor 1 scores and cluster analysis assignment both contain MMP-8, three separate models were created. The multivariate analysis outcomes are shown in Table 3. Model 1 included MMP-8 levels and clinical variables, Model 2 contained Factor 1 scores and clinical variables and Model 3 comprised cluster assignment and clinical variables. All clinical variables included in analysis remained significantly associated with systolic function across all three models. MMP-8 levels and Factor 1 scores were not predictors of systolic dysfunction, while assignment into Cluster One remained an independent predictor of systolic dysfunction (OR 2.74 [95% CI 1.04–7.23], *p* = 0.042).

**Table 3:** Multivariate analysis for systolic function population

Risk factor	Model 1 OR (95% CI)	*P* value	Model 2 (95% CI)	*P* value	Model 3 (95% CI)	*P* value
Current smoker	**0.19 (0.04–0.88)**	**0.034**	**0.19 (0.04–0.88)**	**0.034**	**0.19 (0.04–0.89)**	**0.026**
ACE inhibitor at discharge	**3.15 (1.04–9.51)**	**0.042**	**3.15 (1.04–9.51)**	**0.042**	**3.65 (1.17–11.36)**	**0.035**
Peak hsTnT > 610 (ng/L)	**3.30 (1.28–8.54)**	**0.014**	**3.30 (1.28–8.54)**	**0.014**	**3.14 (1.24–8.00)**	**0.016**
MMP-8 (ng/mL)	3.43 (0.32–36.68)	0.308				
Factor 1 score			0.80 (0.53–1.22)	0.307		
Cluster One assignment					**2.74 (1.04–7.23)**	**0.042**

Model 1 = Current smoking status, prescription of ACE inhibitor at discharge, hs-TnT > median 610 ng/L and MMP-8 concentrations; Model 2 = Current smoking status, prescription of ACE inhibitor at discharge, hs-TnT > median 610 ng/L and Factor 1 score; Model 3 = Current smoking status, prescription of ACE inhibitor at discharge, hs-TnT > median 610 ng/L and Cluster One assignment

Abbreviations: *ACE* angiotensin-converting-enzyme; *hs* high-sensitivity; *MMP-8* matrix metalloproteinase-8; *hsTnT* high-sensitivity Troponin T

Significant p-values are bolded (*p* < 0.05)

## Discussion

In this study, we examined the utility of combining biomarkers to predict the development of systolic dysfunction in a cohort of 120 AMI patients. On univariate analysis, MMP-8 levels, Factor 1 scores and cluster analysis partitioning were associated with the development of impaired systolic function following AMI alongside ACE inhibitor medication at discharge, peak hsTnT levels and current smoking. When multivariate analysis was performed, we demonstrated that Cluster One assignment alongside clinical variables was an independent predictor of impaired systolic function development within 1 year of AMI.

The only ECM biomarker associated with the development of impaired systolic function upon univariate analysis was MMP-8. A previous study conducted by Fertin et al. [27] demonstrated that acute MMP-8 levels were an independent predictor of LV remodelling, defined as a > 20% increase in LV end-diastolic volume, within 1 year of AMI. While LVEF was not directly assessed, MMP-8 levels were also independently associated with the development of cardiovascular death and hospitalisation for heart failure [14], indicating a link between increased MMP-8 levels and LV dysfunction. However, Nilsson et al. [28] has previously demonstrated no association between acute MMP-8 levels and LVEF at 4 months post-MI. These inconsistencies are common throughout the literature, and when LVEF is assessed as the endpoint for LV dysfunction, discordant results are observed for MMP-2, MMP-9 and TIMP-1 [28, 29] [11, 13]. Potentially, opportunistic biomarker testing and timing of LV measurement are important factors influencing these inconsistencies. Additionally, there is no standardisation within the literature to assess LV remodelling and individual studies employ different imaging metrics to asses LV function. In this study, LVEF was used as a global index for systolic function. While this is a common metric, we acknowledge that using alternative LV function endpoints may alter the findings of this study.

Upon multivariate analysis, MMP-8 levels did not remain significantly associated with the development of impaired systolic function. Potentially, these findings, alongside inconsistencies within the literature, suggest that single biomarker analysis is insufficient for capturing the complex pathophysiological processes that comprise adverse LV remodelling. Instead, investigation into more complex methodologies for combining biomarkers that represent cardiac remodelling processes warrants further investigation.

We have previously investigated the clinical utility of combining biomarkers using EFA and cluster analysis [16]. In this study, we applied these techniques to the patient cohort. EFA analysis created a two-factor solution. Factor 1 was composed of MMP-8, MMP-9, MMP-3 and TIMP-1. MMP-8 had the highest loading on this factor and this suggests most of the variance for Factor 1 is captured by MMP-8 levels. Factor 2 comprised of MMP-3, MMP-2, TIMP-1 and PINP, which all demonstrated mid-range positive factor loadings. Factor scores can be generated as a mechanism to study the relationship between clinical outcome and EFA [18]. Factor 1 scores were slightly elevated in patients with impaired systolic function compared to patients with preserved function on univariate analysis. No differences were observed for Factor 2 scores. These findings suggest that biomarkers that make up Factor 1 may more appropriately capture LV remodelling processes and were taken forward for cluster analysis. Cluster analysis separated patients into a two-cluster solution. Patients in Cluster One were more likely to be males, had higher levels of peak hsTnT and were more likely to have impaired systolic function compared to patients in Cluster Two.

When both combined biomarker strategies were assessed using multivariate analysis, Cluster One assignment, but not Factor 1 scores, was independently associated with the development of impaired systolic function within 1 year of AMI. This is of interest, as both methodologies included the same input data as follows: MMP-3, MMP-8, MMP-9 and TIMP-1. Thus, we demonstrate that our combined biomarker analysis methods were not equal in their capacity to predict the development of systolic dysfunction. Differences in predictive power could be associated with the underlying statistical principles of each test and their practical purpose. Cluster analysis is well established in the literature as a methodology for combining variables and examining the relationship between cluster phenotype and outcome [30, 31]. While EFA scores have also been used in this context with success [32, 33], the underlying principle of this methodology is to examine the structure and relationship between variables, and potentially this may reduce the power of EFA for outcome analysis. Regardless, findings from this study outline two important points. First, a combined biomarker strategy may more appropriately risk-stratify patients following AMI when compared to single biomarker analysis. Our findings that Cluster One assignment, but not MMP-8 levels, are predictive of systolic dysfunction suggests that important information is captured by the levels of the three additional biomarkers measured in cluster analysis that are not represented by MMP-8 levels alone. Second, care must be taken when deciding on a methodology to combine biomarkers, as some statistical methods may have more power in a clinical setting than others.

In addition to Cluster One assignment, the prescription of ACE inhibitors at discharge, a current smoking status and peak hsTnT levels above the population median remained significantly associated with the development of impaired systolic function upon multivariate assessment. ACE inhibitor prescription is clinically indicated in patients with chronic heart failure or LV systolic dysfunction upon index admission [34] and thus increased uptake in this patient groups is to be expected The findings of a current smoking status being protective from systolic impairment were more surprising. However, these results were likely skewed by a small sample size of 22 patients reporting as current smokers. While hsTnT is a key marker of myocardial necrosis used in the diagnosis of AMI, peak levels of hsTnT are also a surrogate marker of infarct size [35]. As such, hsTnT levels have been linked to risk of development of systolic dysfunction [35, 36]. In this study, we demonstrated that patients with peak hsTnT greater than 610 ng/L were approximately three times more likely to develop impaired systolic function when compared to patients with lower levels. Our multivariate models suggest that ECM cluster analysis provided additional information that was independent of hsTnT levels regarding risk of systolic dysfunction. ECM biomarkers and hsTnT were assessed separately in this study in order to examine the clinical utility of ECM biomarkers alone to risk-stratify patients following AMI. However, future studies could investigate the benefit of combining hsTnT and circulating biomarkers for risk prediction. Indeed, recent findings have suggested that multi-marker approaches that combine synergistic pathways may be superior at predicting major adverse cardiovascular outcomes following AMI [37]

There are some limitations that should be acknowledged. Timing for echocardiography was not standardised for patients, and this may influence measures of LV remodelling. The median time to echocardiography for preserved and impaired systolic function was 143 (IQR 80–245) days and 163 (IQR 91–296) days, respectively. Despite these numerical differences between patient groups, no statistical differences were observed in timing for echocardiography. Additionally, the median time to echocardiography was approximately 5 months. While this is a common time point recorded in the literature to assess LV remodelling [38, 39], it may be a limitation for measuring advanced remodelling processes. It is important to acknowledge that these biomarkers are not cardiac specific and can be altered by systemic physiological activity. For example, PINP is a biomarker of collagen type I synthesis that is not specific to myocardial tissue alone. However, we have selected an optimal panel of ECM biomarkers based on previous testing that suggested these biomarkers were altered in AMI patients compared to healthy volunteers [19]. This study comprises a moderate sample size and is explorative in nature. Findings from this study should be assessed in a prospective multicentre cohort of AMI patients to ensure validation of these results, and ideally such a study would have a greater number of patients to ensure adequate statistical precision when using multi-marker approaches. Assessing power in a multi-marker study remains challenging when using cluster analysis, as standard power calculations are not appropriate. The sample population included in this study were clinically indicated to receive echocardiogram measurements at follow-up appointments, potentially biasing the population. However, more patients were shown to have preserved systolic function than impaired systolic function. Finally, there is no single standardised methodology within the literature to validate cluster size. In this study, we utilised the NbClust package in R to validate cluster partitioning, with a specific focus on the following two commonly used indices that assess cluster fit: Average Silhouette Width and the ‘Elbow’ method.

In conclusion, we demonstrate that combining ECM biomarkers is useful for predicting the development of impaired systolic function within 1 year of AMI and may provide greater prognostic utility compared to single biomarker analysis. Further research is required to establish the best methodology for combining biomarkers in a clinical setting.

## Supplementary Information

Below is the link to the electronic supplementary material.

Supplementary file1 (JPG 210 KB) The average silhouette width (y-axis) calculates the tightness and separation of a cluster, and is compared across a range of generated clusters (x-axis). A higher value indicates that objects are well matched to their cluster and poorly matched to neighbouring clusters. The highest average silhouette value is shown with a dotted line. This graph was generated on R version 4.0.2 and visualised using the NbClust and Factoextra package

Supplementary file2 (JPG 204 KB) The ‘within sum of squares’ or ‘elbow’ methodology calculates the sum of squares error (y-axis) for a range of clusters (x-axis) in order to determine the optimal cluster fit. This visual methodology looks for a sharp bend (or elbow) in the graph, and is marked with a dotted line. This graph was generated on R version 4.0.2 and visualised using the NbClust and Factoextra package

## Abbreviations

LV: Left ventricular
AMI: Acute myocardial infarction
LVEF: Left ventricular ejection fraction
HFrEF: Heart failure with reduced ejection fraction
ECM: Extracellular matrix
PINP: N-terminal type I procollagen
MMP: Matrix metalloproteinase
TIMP: Tissue inhibitors of matrix metalloproteinase
GRACE: Global registry of acute coronary events
EFA: Exploratory factor analysis
ACS: Acute coronary syndromes
hsTnT: High-sensitivity Troponin T
ASE/EACVI: American SOCIETY OF ECHOCARDIOGRAPHY AND THE EUROPEAN ASSOCIATION OF CARDIOVASCULAR IMAGING
KMO: Kaiser–Meyer–Olkin
ACE: Angiotensin-converting enzyme
