# Supplementary figures and images for: Cluster analysis of extracellular matrix biomarkers predicts the development of impaired systolic function within 1 year of acute myocardial infarction
Source: Heart Vessels. 2022 Jul 27;37(12):2029–38. doi: 10.1007/s00380-022-02118-8 (PMC9579085; doi:10.1007/s00380-022-02118-8)

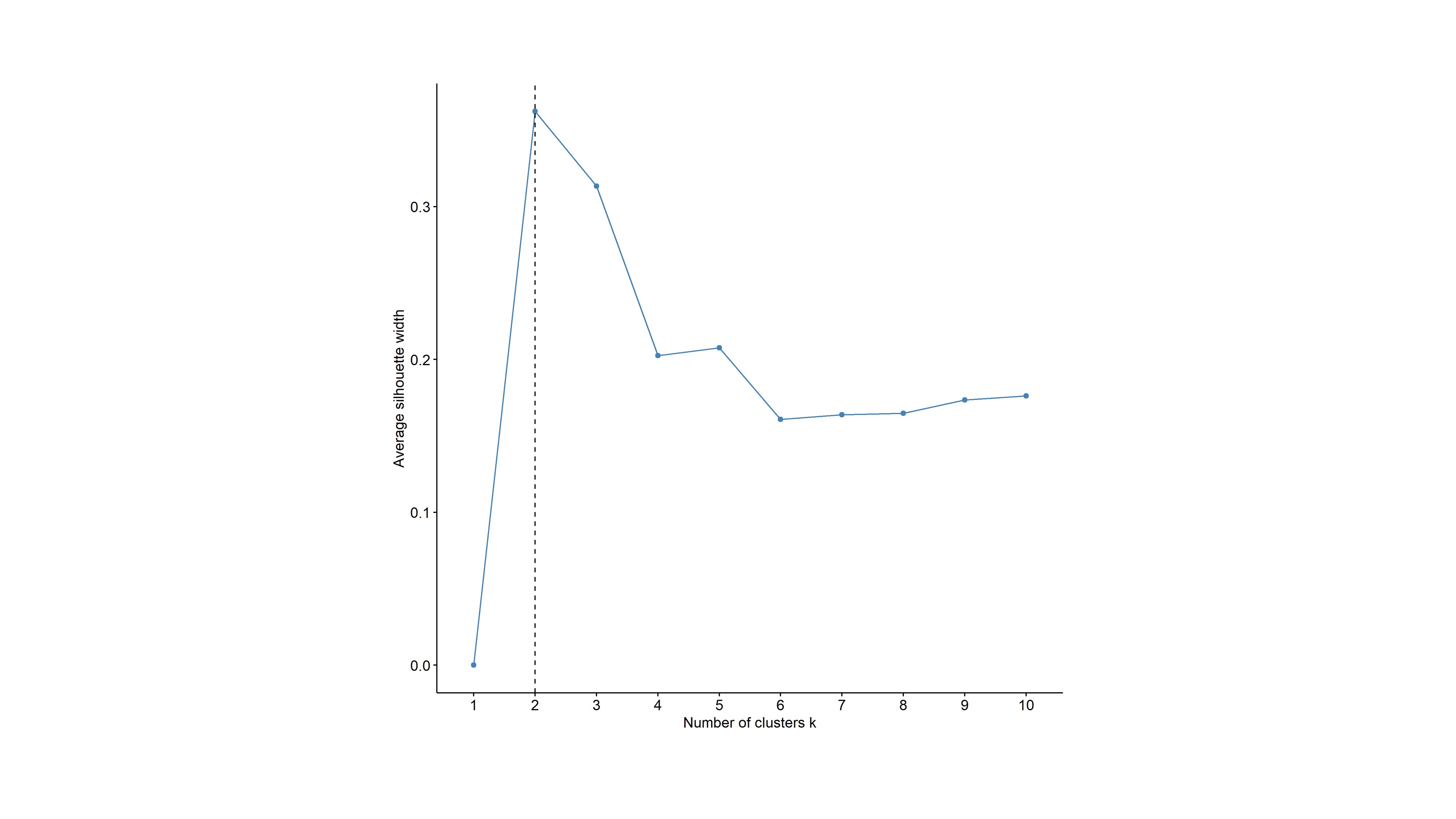

Supplement: Supplementary file 1 — Supplementary file1 (JPG 210 KB) The average silhouette width (y-axis) calculates the tightness and separation of a cluster, and is compared across a range of generated clusters (x-axis). A higher value indicates that objects are well matched to their cluster and poorly matched to neighbouring clusters. The highest average silhouette value is shown with a dotted line. This graph was generated on R version 4.0.2 and visualised using the NbClust and Factoextra package [file 380_2022_2118_MOESM1_ESM.jpg]

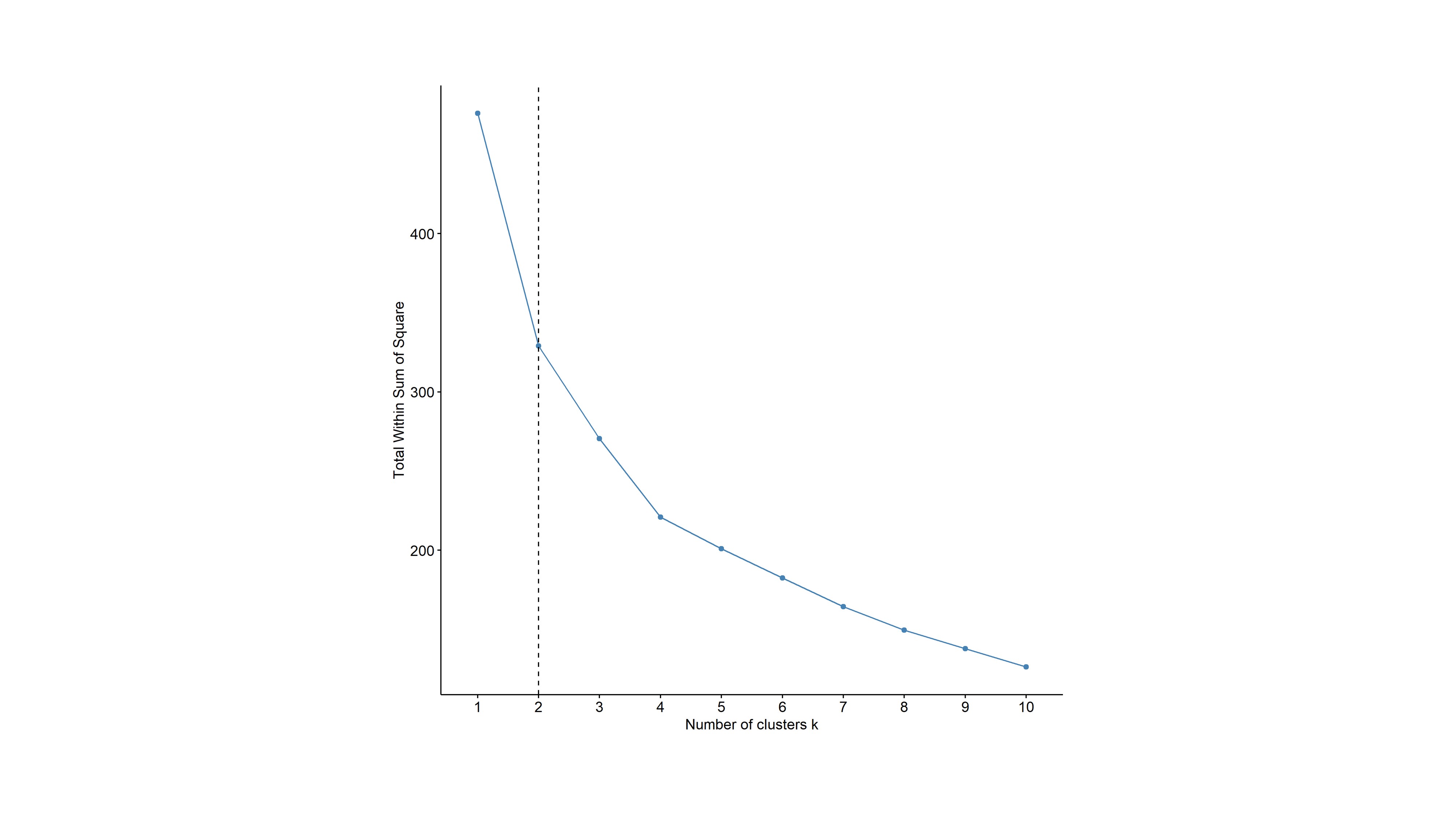

Supplement: Supplementary file 2 — Supplementary file2 (JPG 204 KB) The ‘within sum of squares’ or ‘elbow’ methodology calculates the sum of squares error (y-axis) for a range of clusters (x-axis) in order to determine the optimal cluster fit. This visual methodology looks for a sharp bend (or elbow) in the graph, and is marked with a dotted line. This graph was generated on R version 4.0.2 and visualised using the NbClust and Factoextra package [file 380_2022_2118_MOESM2_ESM.jpg]
